# Performance of Risk-Based Criteria for Targeting Acute HIV Screening in San Francisco

**DOI:** 10.1371/journal.pone.0021813

**Published:** 2011-07-06

**Authors:** Shelley N. Facente, Christopher D. Pilcher, Wendy E. Hartogensis, Jeffrey D. Klausner, Susan S. Philip, Brian Louie, Katerina A. Christopoulos, Teri Dowling, Grant N. Colfax

**Affiliations:** 1 Facente Consulting, San Francisco, California, United States of America; 2 San Francisco General Hospital HIV/AIDS Division, University of California San Francisco, San Francisco, California, United States of America; 3 San Francisco Department of Public Health, STD Prevention and Control, San Francisco, California, United States of America; 4 San Francisco Department of Public Health Microbiology Laboratory, San Francisco, California, United States of America; 5 HIV Prevention Section, San Francisco Department of Public Health, San Francisco, California, United States of America; University of Sao Paulo, Brazil

## Abstract

**Background:**

Federal guidelines now recommend supplemental HIV RNA testing for persons at high risk for acute HIV infection. However, many rapid HIV testing sites do not include HIV RNA or p24 antigen testing due to concerns about cost, the need for results follow-up, and the impact of expanded venipuncture on clinic flow. We developed criteria to identify patients in a municipal STD clinic in San Francisco who are asymptomatic but may still be likely to have acute infection.

**Methods:**

Data were from patients tested with serial HIV antibody and HIV RNA tests to identify acute HIV infection. BED-CEIA results were used to classify non-acute cases as recent or longstanding. Demographics and self-reported risk behaviors were collected at time of testing. Multivariate models were developed and preliminarily evaluated using predictors associated with recent infection in bivariate analyses as a proxy for acute HIV infection. Multivariate models demonstrating ≥70% sensitivity for recent infection while testing ≤60% of patients in this development dataset were then validated by determining their performance in identifying acute infections.

**Results:**

From 2004–2007, 137 of 12,622 testers had recent and 36 had acute infections. A model limiting acute HIV screening to MSM plus any one of a series of other predictors resulted in a sensitivity of 83.3% and only 47.6% of patients requiring testing. A single-factor model testing only patients reporting any receptive anal intercourse resulted in 88.9% sensitivity with only 55.2% of patients requiring testing.

**Conclusions:**

In similar high risk HIV testing sites, acute screening using “supplemental” HIV p24 antigen or RNA tests can be rationally targeted to testers who report particular HIV risk behaviors. By improving the efficiency of acute HIV testing, such criteria could facilitate expanded acute case identification.

## Introduction

In the U.S., HIV antibody testing (using either a rapid test or a laboratory-based enzyme immunoassay (EIA) for antibody screening) remains the most widely used approach to diagnosing HIV infection [1]. However, even the most sensitive antibody tests on the U.S. market today are unable to detect most infections until approximately one month after the onset of infection, as antibodies have not yet been generated in sufficient abundance to trigger a positive reaction to the test. Prior to 2009, guidelines for HIV testing in the U.S. [2] therefore recommended supplemental HIV RNA testing for persons with a suspected acute retroviral syndrome (based on report of both recent high-risk behavior and compatible clinical symptoms).

**Figure 1:**
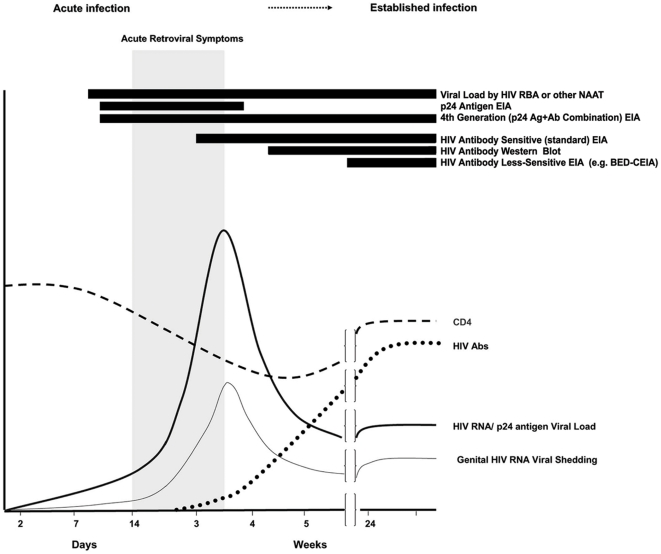
Performance of various HIV assays with respect to rise and fall of CD4 count, HIV antibodies, HIV viral load, and viral shedding over time after infection. Black bars to the left of assay names indicate ability of that assay to detect HIV infection.

Recent data from U.S. testing programs have shown that many HIV infected people who seek HIV testing do so during the initial, antibody-negative acute phase of their infection, due to concern over a specific risk incident, repeated high risk behavior, or occasionally due to symptoms [3]–[6]. This can lead to negative HIV antibody results in many cases of true HIV infection. Missing the diagnosis of acute HIV infection in such antibody-negative, HIV infected individuals is particularly concerning due to the very high potential for sexual HIV transmission that is associated with the initial acute phase of infection [7]–[9]. To address the HIV prevention challenge inherent in identifying HIV testers with acute HIV infection, the U.S. Centers for Disease Control and Prevention (CDC) and Association of Public Health Laboratories (APHL) have issued preliminary algorithms [10] recommending that individuals who have symptoms of acute retroviral infection or report recent high-risk exposure undergo supplemental testing using an assay capable of detecting either HIV RNA or HIV p24 antigen to rule out acute HIV infection (See Figure 1). Accordingly, simply scheduling a future appointment to retest high-risk persons at a later time using less expensive EIA tests is insufficient in high-prevalence areas; targeting RNA or p24 antigen testing as described in this paper allows newly-infected individuals in the initial acute phase to cease behaviors which could unwittingly transmit the virus to sexual partners, as well as to seek medical care very close to the time of infection.

There is a trade-off between the cost and effort needed to identify acute HIV infections and the number of acute infections identified. Specifically, the HIV RNA or HIV p24 antigen tests necessary to identify acute HIV infection can introduce additional cost and complexity to HIV testing algorithms. For some HIV testing sites, especially those using point-of-care rapid HIV testing, simply collecting a tube of blood to allow HIV RNA or p24 antigen testing from every person could create major cost and clinic flow problems. For these reasons, HIV RNA or p24 antigen testing is not part of testing at most sites for voluntary HIV counseling and testing, and individuals with hyper-infectious acute HIV infection who do seek testing are commonly given “negative” HIV test results [4]–[6].

In an attempt to reconcile logistic and cost concerns with prevention priorities, some public health programs have expressed interest in the ‘targeted’ use of acute HIV testing. One study in North Carolina [11] found that testing for acute HIV infection could have been effectively targeted by limiting the use of HIV RNA or p24 antigen testing to “high risk” clinics (e.g. dedicated HIV counseling and testing sites, STD clinics, jails and public health field investigations), and to geographic areas with substantial prevalence of HIV infection (counties reporting >1 new HIV case per 2 years). Sherlock and colleagues completed a survey of current RNA pooling practice in the U.S. in 2007 [12], reviewing practices at all publicly-funded acute HIV detection programs. At that time, only 7 State, County, or City HIV testing programs offered pooled HIV RNA testing and only one (Seattle-King County) offered targeted testing, by simply restricting the eligible population to men who have sex with men (MSM). To date, neither the preliminary CDC/APHL guidelines nor the scientific literature provide additional guidance as to which HIV testers in developed world settings with substantial rates of HIV may actually be at risk of acute HIV infection.

To address this critical gap in knowledge, the present study was performed to develop and evaluate formal criteria for targeting individuals at high risk of acute HIV infection among patients presenting for HIV testing at the San Francisco City Clinic, a municipal STD clinic and site for HIV voluntary counseling and testing in San Francisco, California. This analysis was attempted to systematically determine criteria that would greatly reduce missed acute infections while simultaneously minimizing the proportion of overall clinic patients tested, reducing cost and burden on clinic flow and improving the efficiency of a hypothetical supplemental (HIV RNA/p24 antigen) program. These criteria could then be used by HIV testing clinics wishing to offer a targeted supplemental testing program to improve the effectiveness of HIV testing services.

## Methods

Study participants included all patients who came into the San Francisco City Clinic (SFCC) for HIV testing between January 2004 and December 2007. SFCC is a municipal sexually transmitted disease clinic run by the San Francisco Department of Public Health that offers free, confidential HIV testing for youth and adults in San Francisco. Beginning in October 2003, SFCC has offered screening for acute HIV infection by testing HIV antibody-negative specimens for HIV RNA, using a specimen pooling approach to reduce cost and maximize efficiency. (Using this method, aliquots of 10 HIV antibody-negative specimens are pooled and then tested for HIV-1 RNA [Versant HIV-1 RNA 3.0 Assay, Bayer Corp., Berkeley, CA, USA or Abbott m2000 RealTime HIV-1 Assay, Abbott Molecular, Des Plaines, IL, USA]; if a pooled sample is HIV RNA-positive, each specimen from the pool is then tested individually to determine the HIV-infected individual. This pooling method was chosen after previous validation in San Francisco [5]. Pool size represents a compromise between the maximum cost-efficiency of large pools and the optimal sensitivity and turnaround time with smaller pools [13]. The theoretical limit of detection for any of the RNA tests used can be extrapolated by multiplying the test sensitivity as stated in the package insert by the factor of dilution. For example, the Versant HIV-1 RNA 3.0 Assay has a stated limit of detection of 75 copies [14]; with a .10 dilution the theoretical limit of detection is 750 copies. Similarly, the theoretical limit of detection for the Abbott m2000 RealTime HIV-1 Assay was 400 copies with this pooling strategy [15].) During the study period, HIV antibody testing was performed using one of several FDA-approved antibody tests, including rapid point-of-care HIV tests [OraQuick *ADVANCE*, OraSure Technologies, Inc., Bethlehem, PA, USA] and HIV antibody-only immunoassays [Vironostika HIV-1 Microelisa, BioMérieux Inc., Durham, NC, USA or Genetic Systems HIV-1/2 plus O EIA, BioRad Laboratories, Redmond, WA, USA]. Individuals with antibody negative but HIV RNA positive results were re-tested on subsequent samples using an IgM-sensitive (3^rd^-generation) antibody immunoassay [Genetic Systems HIV-1/2 plus O EIA, BioRad Laboratories, Redmond, WA, USA] and Western blot [Genetic Systems HIV-1 Western Blot, BioRad Laboratories, Redmond, WA, USA] or Immunofluorescence Assay (IFA) [Fluorognost HIV-1 IFA, Sanochemia Pharmazeutika AG, Vienna, Austria]. In addition to this clinical HIV testing, in most cases individuals with HIV antibody-positive test results had blood plasma submitted for incidence testing using the BED Capture Enzyme Immunoassay (BED-CEIA) [Sedia Biosciences Corp., Portland, OR, USA], which is used to classify antibody-positive individuals with regard to having recent (<6 months' duration) or longstanding HIV infection for surveillance purposes.

In this analysis, individuals were classified as having acute HIV infection, likely recent HIV infection or longstanding (non-acute, non-recent) infection based on results of the complete clinical and surveillance testing algorithm. Cases were classified as acute HIV infection if individual testing for HIV RNA was repeatedly reactive but results of the Western blot or IFA were negative or indeterminate [16]. Cases with confirmed positive antibody testing but with a BED-CEIA normalized optical density (ODn)<0.8 were classified as recent HIV infection. Cases with a BED-CEIA ODn>0.8 were classified for the purposes of analysis as having longstanding HIV infection. Self-reported demographic and behavioral risk factors were systematically collected during the initial clinic visit. All procedures were conducted as part of standard clinical and public health practice and all data analysis was done on de-identified data. As these were de-identified public health records undergoing retrospective analyses for public health improvement purposes, no informed consent specific to this study was sought from participants, and this study was considered exempt from human subjects considerations in accordance with the Code of Federal Regulations, Title 45.

This cross sectional analysis utilized an adaptation of the approach used by Miller *et al.* [17] in North Carolina and Powers *et al.* [18] in Malawi. For an initial model development phase, a series of candidate models were developed for the testing population using the outcome of having recent HIV infection. Actual performance of these candidate models in identifying individuals with acute HIV infection was validated using the smaller number of acute HIV infection outcomes in a second analytic step. Model development and validation procedures used a common set of “controls” (that is, patients testing both HIV antibody and RNA negative). Patients with longstanding (non-acute, non-recent) HIV infection were excluded from model development analyses.

It is expected that many individuals testing at a location such as a municipal STD clinic will repeatedly test, sometimes multiple times within one year; as such it is imperative that we assess performance of multiple criteria within a population of repeat testers. As we were attempting to validate the practical application of targeting criteria based on risk behaviors specific to the pre-test interval, each testing interaction for a repeating individual within our dataset was treated as a unique testing encounter.

In bivariate analyses, potential associations between HIV infection status and demographic and risk behavior were tested using Pearson's chi square or Fisher's Exact test where expected cell values were less than 5. Those characteristics that reached p<0.2 in bivariate analyses were then used as candidate predictors in multivariate models during model development using a manual backward selection procedure. Because there were several ways to consider the risk of anal intercourse including a) *any* vs. *specifically receptive* intercourse, and b) *with* vs. *without* consistent condom use, separate but similar groups of models were created with different definitions of this risk factor as one of the input variables.

With the goal of creating a simple set of criteria for RNA testing to streamline clinical decision-making, the same collections of variables were then used to create simple checklists. In this checklist approach, having a specified minimum number of characteristics from a list would determine the need for RNA testing. Performance of the checklists was assessed in terms of sensitivity, specificity, and proportion of patients in which RNA testing was indicated. Because the San Francisco HIV epidemic has predominantly affected MSM, a modified checklist approach was further considered, in which RNA testing would be indicated only for MSM with one or more additional characteristics from a checklist. Finally, RNA testing was considered for anyone who mentioned engaging in any anal intercourse, or specifically receptive anal intercourse alone (in separate models).

Models with ≥70% sensitivity to detect recent infection (indicating potentially adequate sensitivity) and that resulted in RNA testing of ≤60% of patients (indicating a reduction in the proportion tested greater than the reduction in sensitivity) were chosen for further validation. These selected models were then validated using acute infection cases, and performance characteristics including area under the receiver operator characteristic (ROC) curve were calculated for all selected models.

## Results

From 2004 though 2007, there were 12,622 tests for HIV at the San Francisco City STD Clinic. Of these, 233 (1.9%) people had longstanding HIV infection and were not included in model development. The study population included 137 (1.1%) people with recent HIV infection, 36 (0.29%) with acute infection, and 12,216 uninfected people who comprised the comparison group for all analyses. Because of missing demographic data, 3 people were omitted from model development, all of whom had tested HIV negative.

The median age of testers was 32 years (IQR: 26–39 years). Fifty four percent of patients were white, 20% were Latino, 10% Black, and the remaining 16% Asian, Native American, or multiethnic (the latter were grouped together because of low rates of recent infection compared with other demographic groups). The study population was 85% male, and 75% MSM. Four percent reported a history of injection drug use. Characteristics that were significantly associated with recent infection in bivariate analyses included race/ethnicity, gender, sexual orientation (self identified as gay/lesbian/bisexual/queer/questioning (GLBQQ) or “other”, or defined by behavior as MSM), history of anal intercourse (using any definition including receptive or insertive or both, and with or without consistent condom use, and regardless of gender), sex with a known HIV positive partner, history of injection drug use, and recent history of a sexually transmitted infection (Table 1). Race/ethnicity was not included in multivariate models because of potential misunderstanding and misrepresentation of the race/ethnicity variable, as well as ethical concerns about the use of race or ethnicity to determine eligibility for a particular set of healthcare services [19]–[20]. Gender was also left out of multivariate models because of covariance with MSM. Age was included in model development as a final *a priori* predictor, but was eventually dropped from the models because it proved to be not statistically significant, unlike the other candidate predictors. (Note: results of models were similar whether self-identified sexual orientation (GLBQQ/Other) or behaviorally-defined MSM was used as a starting point. Models with MSM are presented here.)

**Table 1:** Characteristics, prevalence of longstanding and recent HIV infection, and associations with recent HIV infection among persons undergoing HIV testing at the San Francisco City Clinic from 2004–2007 (development data; n = 12622).

			Longstanding infection	Recent infection		
Characteristic	Levels	Population Frequency % (n/N)	Prevalence % (n/N)^1^	Prevalence % (n/N)^2^	OR (95% CI)	p-value
Total population		100% (12622)	1.9% (233/12449)	1.1% (137/12353)		
Age^3^	< = 40 years	78.1% (9852/12619)	2.1% (57/2736)	1.2% (112/9646)	1.26 (0.81–1.95)	0.30
	>40 years	21.9% (2767/12619)	1.8% (176/9710)	0.9% (25/2704)	Reference	
Race/ethnicity	Black	10.3% (1303/12606)	2.6% (33/1278)	1.5% (19/1264)	1.45 (0.87–2.43)	0.014
	Latino	19.9% (2503/12606)	2.7% (65/2457)	1.5% (37/2429)	1.47 (0.99–2.20)	
	Mixed/Other	16.1% (2034/12606)	1.6% (32/2019)	0.6% (12/1999)	0.58 (0.31–1.06)	
	White	53.7% (6766/12606)	1.5% (103/6679)	1.0% (69/6645)	Reference	
Gender	Male	85.2% (10749/12616)	2.1% (223/10578)	1.3% (135/10490)	11.06 (2.73–44.70)	<0.0001
	Trans/Other	1.3% (165/12616)	3.6% (6/165)	0% (0/159)	0 (undefined)	
	Female	13.5% (1702/12616)	0.2% (4/1700)	0.1% (2/1698)	Reference	
Sexual Orientation	GLBQQ/Other^4^	75.2% (9439/12554)	2.4% (219/9277)	1.4% (126/9184)	4.30 (2.26–8.20)	<0.0001
	Straight	24.8% (3115/12554)	0.4% (13/3105)	0.3% (10/3102)	Reference	
MSM	MSM	75.0% (9472/12622)	2.3% (217/9309)	1.4% (127/9219)	4.36 (2.29–8.32)	<0.0001
	Not MSM	25.0% (3150/12622)	0.5% (16/3140)	0.3% (10/3134)	Reference	
Anal intercourse (AI)^5^	Any AI	71.6% (9038/12622)	2.2% (198/8883)	1.4% (121/8806)	3.07 (1.82–5.19)	<0.0001
	No AI	28.4% (3584/12622)	1.0% (35/3566)	0.5% (16/3547)	Reference	
Unprotected anal intercourse (uAI)^5^	Any uAI	43.7% (5515/12622)	2.8% (148/5389)	1.9% (99/5340)	3.47 (2.38–5.05)	<0.0001
	No uAI	56.3% (7107/12622)	1.2% (85/7060)	0.5% (38/7013)	Reference	
Receptive anal intercourse (RAI)^5^	Any RAI	55.8% (7045/12622)	2.5% (173/6907)	1.6% (106/6840)	2.78 (1.86–4.16)	<0.0001
	None	44.2% (5577/12622)	1.1% (60/5542)	0.6% (31/5513)	Reference	
Unprotected receptive anal intercourse (uRAI)^5^	Any uRAI	31.2% (3938/12622)	3.2% (123/3828)	2.2% (85/3790)	3.76 (2.65–5.31)	<0.0001
	None	68.8% (8684/12622)	1.3% (110/8621)	0.6% (52/8563)	Reference	
Injection drug use^5^	Any IDU	4.2% (531/12622)	2.3% (12/518)	2.5% (13/519)	2.43 (1.36–4.33)	0.002
	None	95.8% (12091/12622)	1.9% (221/11931)	1.1% (124/11834)	Reference	
Any sex with known HIV+ partner^5^	Yes	17.2% (2168/12622)	3.2% (68/2102)	2.5% (51/2085)	2.97 (2.09–4.21)	<0.0001
	No	82.8% (10454/12622)	1.6% (165/10347)	0.8% (86/10268)	Reference	
Recent STD^6^	Yes	32.9% (4153/12606)	2.4% (96/4077)	1.4% (58/4039)	1.52 (1.08–2.14)	0.016
	No	67.1% (8453/12606)	1.5% (121/8356)	1.0% (79/8314)	Reference	

1 Longstanding HIV prevalence: prevalence of longstanding infection over the entire study period (2004–2007) (note: recent and acute infections were excluded from the denominator).

2 Recent HIV prevalence: prevalence of recent infection over the entire study period (2004–2007) (note: acute and longstanding infections were excluded from the denominator).

3 Age, median (IQR): 32 yrs (26–39).

4 GLBQQ/Other group includes gay, lesbian, bisexual, queer, “don't know” (questioning), and “other”.

5 All risk behaviors occurring since time of last HIV test or within the past 2 years, whichever is shorter.

6 Sexually transmitted infection diagnosed since time of last HIV test or within the past 2 years, whichever is shorter.

Sets of logistic models with different definitions of anal intercourse followed similar patterns in the backwards selection procedure, dropping age and STD history for a reduced model with anal intercourse, history of a known HIV positive partner, history of injection drug use, and any reported male-male sexual behavior as predictors. Unprotected receptive anal intercourse was more strongly associated with recent infection than any other definition of anal intercourse, and full and reduced models using unprotected receptive anal intercourse are presented in Table 2. The strongest positive associations with recent infection were with unprotected receptive anal intercourse (OR = 2.82, 95% CI: 1.97–4.04), and any reported male-male sexual behavior (OR = 2.76, 95% CI: 1.42–5.40). Risk of recent infection was also higher for individuals reporting history of a known HIV positive partner (OR = 2.14, 95% CI: 1.49–3.06), and history of injection drug use (OR: 2.19, 95% CI: 1.21–3.98).

**Table 2:** Full multivariable predictive model, and models reduced through backwards selection, for recent HIV infection among persons undergoing HIV testing at the San Francisco City Clinic from 2004–2007 (development data; n = 12350; excludes acute and longstanding infections and n = 3 HIV negative people with missing demographic data).

	Model 1: Full Model			Model 2: Reduced Model		
Characteristic	co-efficient	OR (95% CI)	p-value	co-efficient	OR (95% CI)	p-value
Unprotected RAI						
Any	1.019	2.77 (1.93–3.97)	<0.0001	1.037	2.82 (1.97–4.04)	<0.0001
None		Referent			Referent	
HIV+ Partner						
Any	0.740	2.10 (1.46–3.01)	<0.0001	0.759	2.14 (1.49–3.06)	<0.0001
None		Referent			Referent	
Injection drug use						
Any	0.775	2.17 (1.20–3.94)	0.01	0.785	2.19 (1.21–3.98)	0.01
None		Referent			Referent	
MSM						
Any	1.013	2.75 (1.41–5.38)	0.003	1.016	2.76 (1.42–5.40)	0.003
None		Referent			Referent	
Recent STD						
Any	0.222	1.25 (0.88–1.76)	0.21			
None		Referent				

In addition to creating checklists as a simple tool for clinical decision making about whether to send specimens for RNA testing, creation of a risk score algorithm based on logistic model coefficients was considered, in the manner of Powers and colleagues [17]. However, in this study population, where multivariate adjusted risks and therefore logistic model coefficients were all within a narrow range, this approach effectively reduced to a simple, unweighted checklist approach because predictors were all given equal weight.

Several checklist models met the pre-determined definition of acceptable performance (≤60% patients tested; ≥70% sensitivity for recent infection) and were further validated with respect to the outcome of acute infection (Table 3). One of the best performing checklists was based on Table 2, model 1, and involved RNA testing for anyone with two or more of the following characteristics: male-male sexual behavior, unprotected receptive anal intercourse, history of a known HIV positive partner, history of injection drug use, or recent history of a sexually transmitted infection. This checklist resulted in RNA testing for 49.7% of patients undergoing RNA testing and a sensitivity of 83.3% (95% CI: 67.2–93.6%) to detect acute cases (Table 3, model 1). A checklist that indicated testing only for people reporting male-male sexual behavior who had one or more other key characteristics (unprotected receptive anal intercourse, history of a known HIV positive partner, history of injection drug use, or recent history of a sexually transmitted infection) had similar performance (Table 3, model 3). This latter model resulted in testing 47.6% of patients for HIV RNA and the model had 83.3% (95% CI: 67.2–93.6%) sensitivity to detect acute infection.

**Table 3:** Performance of models selected during the development stage (using recent infection as the outcome), here validated using acute HIV infection cases as the outcome.

Model	Checklist characteristics^1^	Proportion referred for testing (% [n/N])	Sensitivity (95% CI)	Specificity (95% CI)	ROC AUC
Model 1	≥2 from list: MSM, IDU, +partner, uRAI, STI	49.7% (6086/12249)	83.3% (67.2–93.6)	50.4% (49.5–51.3)	0.669
Model 2	≥2 from list: MSM, IDU, +partner, uRAI	37.3% (4573/12249)	75.0% (57.8–87.9)	62.8% (61.9–63.6)	0.689
Model 3	MSM +1 from list: IDU, +partner, uRAI, STI	47.6% (5826/12249)	83.3% (67.2–93.6)	52.5% (51.7–53.4)	0.679
Model 4	Single risk factor: AI	71.2% (8719/12252)	94.4% (81.3–99.3)	28.9% (28.1–29.7)	0.617
Model 5	Single risk factor: uAI	43.0% (5268/12252)	75.0% (57.8–87.9)	57.1% (56.2–58.0)	0.661
Model 6	Single risk factor: RAI	55.2% (6766/12252)	88.9% (73.9–96.9)	44.9% (44.0–45.8)	0.669
Model 7	Single risk factor: uRAI	30.4% (3730/12252)	69.4% (51.9–83.7)	69.7% (68.8–70.5)	0.696
Model 8	uRAI or ≥2 from list: MSM, IDU, +partner, STI	51.1% (6255/12249)	83.3% (67.2–93.6)	49% (48.1–49.9)	0.662

**All models are based on acute HIV infection among persons undergoing HIV testing at the San Francisco City Clinic from 2004–2007 (validation data; n = 12252; excludes recent and longstanding infections).**

1 **Key to checklists:** MSM (person is a man who had sex with male partner(s)); IDU (any history of injection drug use); +partner (person had a partner known to be HIV positive); uRAI (engaged in unprotected receptive anal intercourse); STI (had a sexually transmitted infection within the past 2 years or since the last HIV test); AI (engaged in any anal intercourse, regardless of sex or gender); uAI (engaged in unprotected anal intercourse, regardless of sex or gender); RAI (engaged in receptive anal intercourse, regardless of sex or gender); uRAI (engaged in unprotected receptive anal intercourse, regardless of sex or gender).

Finally, simplified models were evaluated, in which different risk factors were considered as single criteria for supplemental testing; results are shown in Table 3. Two of these simple models—focusing on any unprotected anal intercourse or any receptive anal intercourse as a single criterion for testing—performed nearly as well as the more complicated checklists. Using unprotected anal intercourse alone (Table 3, model 5) resulted in 43.0% of patients tested and sensitivity of 75.0% (95% CI: 57.8–87.9%) for acute infection. Using any receptive anal intercourse alone (Table 3, model 6) resulted in 55.2% of patients being tested and a sensitivity of 88.9% (95% CI: 73.9–96.9%) for acute infection.

Results of the ROC area under the curve (AUC) analysis for the final models (Table 3) highlighted that the performance of our final models were largely similar. However, two of the models with the greatest ROC AUC had substantially lower sensitivity for acute infection (69.4 and 75.0%) than other candidate models. Ultimately, this parameter was not found to be useful in deciding which model had the best overall performance.

## Discussion

This San Francisco, STD clinic-based study found that that simple criteria based on behavioral risk factors for acute HIV infection performed well as clinical prediction rules to identify individuals with a particularly high risk of acute HIV infection. Results suggest that if such criteria were used to target the use of HIV RNA or p24 antigen testing at similar sites, one could reduce the need for this type of testing by about half, while still identifying as many as 80% to 90% of acute HIV infections. These results show that even in high prevalence/high risk areas, additional criteria related to self-report of HIV risk behavior could be used for highly effective targeting of HIV RNA or p24 antigen-based acute HIV testing—allowing more efficient use of these acute HIV screening tests with only a modest decrease in acute case finding.

The factors most strongly associated with having acute HIV infection in the STD clinic population were male biological sex, gay identity, male-male sexual behavior, and anal intercourse. Remarkably, even very simple criteria (for example, a single behavioral risk factor such as receptive anal intercourse) performed nearly as well as more complex, multi-variable models. This may be explained by the degree to which significant sexual risk factors for having acute HIV infection co-varied in this clinic population. While it is important to note that no single risk factor necessarily performed as well as the best checklists evaluated in this study, the gains in sensitivity, specificity or testing efficiency associated with the more complex models were modest. The relatively good performance of simple models indicates that in clinic settings where detailed risk factors are not routinely collected, single risk factors could potentially be used effectively to target acute HIV testing.

Missing even a few cases of acute HIV infection is undesirable, and the use of comprehensive testing algorithms for all patients at all sites would offer the best acute case detection. The benefits of criteria for targeting RNA or p24 antigen testing could apply mainly to sites where performing the additional procedures involved for such testing (e.g., phlebotomy) is problematic due to cost, personnel, space, or other factors.

These results are subject to several limitations. When dealing with sensitive, self-reported data, reporting bias may be a factor. Furthermore, a number of data fields had high rates of missing data or inconsistent information, which precluded their use in the analysis. Another potential limitation is the use of BED-CEIA test results for classification of cases as having “recent” or “longstanding” infection, as this test is subject to overestimation of recent infections [21]–[23]. However, the performance of recent infection status (based on BED results) as a population-level proxy measure for acute HIV, when validated against the acute infection outcome, was consistent with the previous findings of Miller, *et al*, who used the Vironostika LS-EIA in North Carolina [24]. In addition, the criteria developed were in a city with an HIV epidemic that is heavily concentrated among gay men [25] and cannot be considered generalizable to other cities with distinct HIV epidemiology. Specifically, so few women were included in the raw data that we did not have sufficient power in this analysis to assess the odds associated with vaginal intercourse or other sexual behaviors related specifically to women. Additionally, because the numbers of transgender people were also extremely low and there were no acute cases among transgender individuals in our dataset, transgenders did not appear in the criteria; however, since epidemiological risk profiles in San Francisco [26] and biological risk factors (e.g. URAI) between trans women and MSM are so similar, it would be reasonable to interpret our findings regarding MSM to also apply to trans women. Finally, no information was collected on the presence or absence of possible viral-syndrome symptoms from testers.

In summary, this analysis found that simple criteria based on self-report of HIV risk behavior could be used to target the use of HIV RNA or p24 antigen-based acute HIV testing, resulting in a substantially reduced volume of supplemental testing in high risk settings, while preserving the ability of a testing program to detect acute HIV infections and ensure accurate HIV case identification.

Given the high likelihood of sexual transmission during the acute phase of HIV infection, detecting acute HIV infections could hypothetically impact the spread of HIV in populations, as described by Powers and colleagues (International AIDS Conference, 2010). However, in this era of reduced resources for HIV prevention, the expansion of efforts to identify acute HIV infections at HIV testing sites will require feasible approaches that can minimize costs and complexity while maximizing acute case detection. Expansion of acute HIV testing services to new areas may be possible with the introduction of simpler assays for acute HIV detection in the U.S., including approved 4^th^ generation immunoassays [27] and rapid tests still in development. Using acute HIV tests with rational criteria for targeting their use offers an important opportunity to meet these goals and potentially influence the course of the HIV epidemic in San Francisco.
